# Clinical and molecular predictors of very late recurrence in oestrogen receptor-positive breast cancer patients

**DOI:** 10.1007/s10549-024-07311-z

**Published:** 2024-05-06

**Authors:** Juliet Richman, Gene Schuster, Richard Buus, Elena Lopez-Knowles, Mitch Dowsett

**Affiliations:** 1 Ralph Lauren Centre for Breast Cancer Research, Royal Marsden Hospital, London, UK; 2 Breast Cancer Now Toby Robins Research Centre, The Institute of Cancer Research, London, UK

**Keywords:** Breast cancer, Oestrogen receptor, CTS5, Very late recurrence

## Abstract

**Background:**

Risk of recurrence from primary ER+ breast cancer continues for at least 20 years. We aimed to identify clinical and molecular features associated with risk of recurrence after 10 years.

**Methods:**

ER+ breast cancers from patients with and without recurrence were analysed with the BC360 NanoString Panel and an 87 gene targeted-exome panel. Frequency of clinical, pathologic and molecular characteristics was compared between cases (recurred between 10 and 20 years) and controls (no recurrence by 20 years) in the Very Late Recurrence (VLR) cohort. Analogous data from METABRIC were examined to confirm or refute findings.

**Results:**

VLR cases had larger tumours and higher node positivity. Both VLR and METABRIC cases had higher clinical treatment score at 5 years (CTS5). There was a trend for fewer *GATA3* mutations in cases in both VLR and METABRIC but no statistically significant differences in mutation frequency. Cell cycle and proliferation genes were strongly expressed in VLR cases. Immune-related genes and cell cycle inhibitors were highly expressed in controls. Neither of these changes were significant after correction for multiple testing.

**Conclusions:**

Clinicopathologic features are prognostic beyond 10 years. Conversely, molecular features, such as copy number alterations, *TP53* mutations and intrinsic subtype which have early prognostic significance, have little prognostic value after 10 years.

**Supplementary Information:**

The online version contains supplementary material available at 10.1007/s10549-024-07311-z.

## Background

Breast cancer patients with oestrogen receptor-negative (ER-) disease that recur, mostly do so within the first 10 years of follow-up [1]. In contrast, ER+ breast cancer has an almost linear risk of recurrence up to 20 years from diagnosis [2]. For ER+ disease, standard clinicopathologic features of early recurrence appear to maintain at least some of their significance for late recurrence and one integrative prognostic tool, the CTS5 (Clinical Treatment Score after 5 years) has been developed to estimate risk between 5 and 10 years after diagnosis in the absence of endocrine treatment beyond 5 years [3]. It is also clear that while some genomic profiling tests maintain some prognostic significance out to 10 years, this differs between the tests [4, 5]. Additionally, over the period between 5 and 10 years after diagnosis, increased recurrences have been reported to occur in patients with tumours with high levels of both oestrogen-responsive and proliferation-associated genes [6, 7].

Over recent years, at 5 years from diagnosis, women have had the option to continue their endocrine therapy (ET) for a further 5 years because of trial data showing the overall benefit of this for reducing recurrence risk [8–11]. This decision is usually made purely on the basis of clinicopathologic features of the primary tumour and patient factors. Given a potentially life-long risk of recurrence and positive data from one clinical trial of endocrine therapy beyond 10 years [11], there remains a need to distinguish those that have a significant risk of recurrence even after completing 10 years of ET.

Despite the long natural history of ER+ breast cancer, little is known about the molecular features that predict for recurrences beyond 10 years. Multiple mechanisms have been suggested to underpin the emergence of subclinical disease from apparent dormancy as clinically evident very late recurrence [12]. The data that support the proposed mechanisms are almost entirely based on observations in non-clinical model systems. A recent publication on data from 3,240 patients with a median follow-up time of 14 years reported different patterns of recurrence according to molecular features but in 80% of cases the data on distant recurrence (DR) were provided by an algorithmic model rather than observation [13].

A difficulty of studying molecular predictors of very late recurrence (VLR) is the protracted follow-up time that is required in order to determine whether a woman is truly recurrence free. Studies therefore almost inevitably need to be retrospective and require the availability of tissues that have been stored for decades. Additionally, to provide adequate statistical power a large initial sample size is needed owing to the relative rarity of the outcome event. Few studies have therefore been conducted but the large breast cancer practise at the Royal Marsden Hospital and its policy of long-term storage of tissue biopsies provided the opportunity to do so.

The aim of this project was to determine whether differences could be identified in gene expression and/or DNA alterations between patients that eventually developed a VLR compared to those with persistent absence of recurrence. To determine the confidence in the results derived, where data availability allowed, we undertook analyses of the METABRIC cohort for comparison [14].

## Methods

### Study participants

This is a retrospective case–control study of female patients with early ER+ breast cancer diagnosed at the Royal Marsden Hospital between 1988 and 1998 with a follow-up of at least 10 years for cases and at least 20 years for controls. Cases were defined as DR beyond ten years from diagnosis and controls were defined as alive and DR free at 20 years from diagnosis. Patients dying from any cause prior to 10 years of follow-up or dying without recurrence in years 10–20 due to non-breast cancer causes were excluded. Other exclusion criteria were ER-negative and HER2-positive disease, patients not treated curatively, and for controls, any DR during follow-up period, DR in years 0–10 and non-invasive pathology. ER-negative samples were excluded based on ER status in the clinical files and ER unknowns were included unless subtype was found to be basal-like after molecular analysis. HER2-positive patients were also excluded after molecular analysis based on *ERBB2* expression and copy number amplifications (CNA). We classified samples as HER2-positive if log2 expression > 12 based on the observed relationship between CNA and expression of ERBB2 and GRB7.

### Samples

DNA and RNA were co-extracted using the Qiagen Allprep FFPE kit from microdissected FFPE sections according to the manufacturer’s instructions (Qiagen, Hilden, Germany). Where the patient had neo-adjuvant treatment, FFPE blocks of the diagnostic cores were used. Quantification was done using high sensitivity RNA and DNA Qubit assays (Thermo Fisher Scientific, Carlsbad, CA).

### DNA sequencing

A targeted-exome panel was designed covering 87 genes (Supplementary Table 1) selected to include genes affected by driver mutations. The panel allowed detection of chromosomal instability across the genome. For detection of copy number variation pooled blood diploid normal controls were used. Preparation of 250 ng DNA and DNA capture were conducted using SureSelect XT Low Input Reagent Kit (Agilent, Santa Clara, CA) and sequencing used the NovaSeq platform (Illumina, San Diego, CA).

### Copy number alteration (CNA) analysis

BWA software (version 0.7.15) was used to align the sequences, any duplicated reads were identified using Picard tools (version 2.8.2) and CNVkit pipeline was used for detection of CNA gains and losses.

### Mutation detection

Mutation detection was conducted using Mutect2 software from GATK (version 4.0.5.1). Mutect2 has an orientation bias filter that helps reduce false positives of C > T caused by long-term storage of FFPE tissue. Ensembl variant effect predictor (VEP) was then used to determine the effect of the detected variants, to annotate the mutations and map them to coding or splicing regions in the genome. Only somatic mutations with a moderate-to-high impact on the translated protein were included in the analysis. These mutations were then manually checked whereby a cut-off of 5% allele frequency with a minimum of 10 mutant allele counts was selected for an aberration to be called a true mutation.

### Gene expression assay

One hundred and fifty ng of RNA was used to measure gene expression using the Breast Cancer 360 panel on the NanoString nCounter platform according to the manufacturer’s instructions (NanoString, Seattle, WA). This panel targets 776 genes involved in multiple different pathways in breast cancer such as proliferation, invasion and those linked with the tumour microenvironment and immune response [15].

### RNA expression analysis

Raw NanoString gene expression data were normalized with NanoStringNorm (version 1.2.0) package [16] using R software (version 3.6.1 [17]). Gene set enrichment analyses were performed according to four sets of gene annotations: Enrichr [18, 19], the gene ontology biological processes annotations [20, 21], KEGG annotations [22] and Hallmarks of cancer [23]. Tumours were classified into one of the intrinsic subtypes (Luminal A, Luminal B, Basal-like and HER2-enriched) based on the PAM50 classifier algorithm [24, 25].

### Other datasets

The METABRIC dataset was used for comparison of copy number, mutations and gene expression [14].

### Statistical analysis

The primary endpoint of the study was proportion of overall CNAs (measured by fraction of genome altered) in VLR versus no recurrence groups for the whole population of cases and controls. Mann–Whitney U test in R software (v 3.6.1) [17] was used to compare overall CNA in cases and controls.

Secondary endpoints were clinical (size, grade, lymph node burden and treatment) and molecular variables (mutations and expression). Mann–Whitney test was used to compare age, tumour size and CTS5 value. Comparison of categorical variables was assessed using *χ*^2^ test (stats package within R software v3.6.1) [17] and comparison of grade was made by *χ*^2^ test for trend.

All analyses were 2-sided with an alpha level of 0.05. Owing to the large number of genes being tested, all analyses were subject to correction for multiple testing using the Benjamini–Hochberg False Discovery Rate (FDR) method.

## Results

### Patients and clinicopathologic data

From an initial list of 1335 patients diagnosed between 1988 and 1998, 194 controls and 96 cases were reviewed on the Electronic Patient Record system (Supplementary Figure 1). After tissue-related exclusions, 50 cases (recurrence between years 10 and 20) and 67 controls (disease free beyond 20 years) had RNA and DNA extracted from the tumour. After further exclusions on the basis of insufficient material, ER-negative or HER2-amplified status, 98 samples (44 cases, 54 controls) had RNA expression and 71 samples (38 cases, 34 controls) had DNA sequencing data. Clinico-pathological parameters are described in Table 1. Median age was 50 with no significant difference between cases and controls. Cases had significantly larger tumours than controls (21 mm vs 16 mm, *p* = 0.01) and a significantly greater proportion of patients with node-positive disease (*p* = 0.0002). A larger proportion of cases were treated with chemotherapy compared to controls (76% vs 42% *p* = 0.0007). The CTS5 was calculated for the subset of cases and controls with all relevant data available and it was significantly higher in cases compared to controls 3.63 versus 2.91, *p* = 0.0003. Histological subtype was evenly distributed between cases and controls with 75% of all cancers being invasive ductal carcinoma. Data on menopausal status were lacking in many patients as these data were rarely codified in older patient record systems. In the overall patient population, 28 patients were pre-menopausal, 27 were post-menopausal and 43 had unknown menopausal status. The most common site of metastasis was bone (55%) followed by liver, lung and nodal tissue. 40% patients had more than one site of metastasis. Local recurrence was more common in cases (42%) compared to controls (34%).

**Table 1:** Baseline characteristics of patients within the VLR study and METABRIC group > 10 yr

	VLR				METABRIC		
	All (*n* = 98)	Cases (> 10 yr R, *n* = 44)	Controls (> 20 yr no R, *n* = 54)	*p* value	Cases (> 10 yr R, *n* = 68)	Controls (> 20 yr noR, *n* = 48)	*p* value
Age (median)	50	48	51	0.23	62	61	0.39
Menopausal status							
Pre	28	14	14	0.59	14	8	0.64
Post	27	11	16		54	40	
Unknown	43	19	24				
Histological subtype							
IDC	75	32 (73%)	43 (79%)	0.27	50 (74%)	30 (63%)	0.33
ILC	14	8 (18%)	6 (11%)		5 (7%)	7 (15%)	
Mixed	2	0 (0%)	2 (4%)		10 (14%)	9 (19%)	
Unknown	7	4 (9%)	3 (6%)		3 (4%)	2 (4%)	
ER positive	82 (84%)	39 (89%)	43 (80%)	0.23	68 (100%)	48 (100%)	NA
ER unknown	16 (16%)	5 (11%)	11 (20%)		0	0	
Tumour size, mm (median)	17.5	21	16	0.01	25	22	0.19
Grade							
Grade 1	9 (9%)	5 (11%)	4 (7%)	0.24	3 (4%)	6 (12%)	0.052
Grade 2	61 (63%)	22 (50%)	39 (73%)		29 (43%)	19 (40%)	
Grade 3	14 (14%)	10 (23%)	4 (7%)		34 (50%)	21 (44%)	
Unknown	14 (14%)	7 (16%)	7 (13%)		2 (3%)	2 (4%)	
Lymph node burden							
Node negative	50 (51%)	13 (30%)	37 (69%)	0.0002 (pos vs neg)	24 (35%)	24 (50%)	0.16
Node positive	38 (39%)	25 (57%)	13 (24%)		44 (65%)	24 (50%)	
Unknown	9 (95%)	6 (13%)	4 (7%)		0	0	
Chemotherapy treated	56 (57%)	33 (76%)	22 (42%)	0.0007	6 (9%)	0 (0%)	NA
Endocrine therapy treated	90 (92%)	42 (95%)	48 (89%)	0.24	68 (100%)	48 (100%)	NA
Duration endocrine therapy, months (median)	60	60	60	0.63	Unknown	Unknown	
Local recurrences n (%)	36	18 (42)	18 (34)		N/A	N/A	
Time to distant recurrence, years (median)	N/A	15	N/A		14	N/A	
Sites of distant recurrence							
Bones	24	24	N/A		N/A	N/A	
Liver	10	10	N/A		N/A	N/A	
Lung/pleura	11	11	N/A		N/A	N/A	
Lymph nodes	11	11	N/A		N/A	N/A	
Other	10	10	N/A		N/A	N/A	
Follow-up, years (median)	N/A	N/A	21		N/A	21.80	
CTS5 value (median)	3.19 (*n* = 81)	3.63 (*n* = 35)	2.91 (*n* = 46)	0.0003	4.09 (*n* = 65)	3.68 (*n* = 46)	0.0010

Sites of distant recurrence include all sites i.e. some patients had more than one

The demographic information for the > 10 yr recurrence and > 20 yr non-recurrence groups METABRIC is also shown in Table 1. The METABRIC dataset also showed a similar trend for differences in tumour size and nodal status and a highly significant difference in CTS5 between the cases and controls. The demographic information for the 0–5 yr and 5–10 yr groups is described in Supplementary Table 2.

**Fig. 1:**
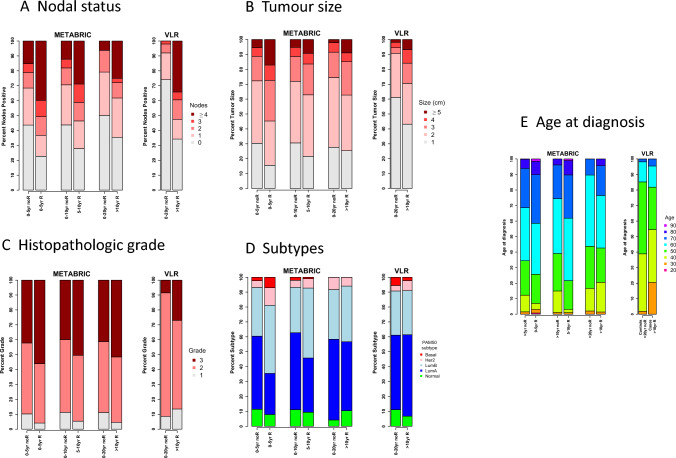
Percentage of cases and controls in METABRIC and VLR **A** nodal status, **B** tumour size, **C** histopathologic grade. **D** Percentage of cases and controls in METABRIC and VLR according to PAM50 subtypes. **E** Percentage of cases and controls in METABRIC and VLR according to the age at diagnosis by decade. Three time intervals after diagnosis are shown for METABRIC: Recurrence (R) in years 0–5 (0–5 yr R) vs no recurrence in years 0–5 (0–5 yr noR); recurrence in years 5–10 (5–10 yr R) vs no recurrence in years 0–10 (0–10 yr noR); recurrence after 10 years (10 yr R) vs no recurrence in years 0–20 yr (0–20 yr noR). *Note* 0–5 yr noR and 0–10 yr noR groups include patients who went on to recur at a later time

The relationship between these clinicopathologic data and risk of DR in VLR and METABRIC is shown in Fig. 1A–E. The data from METABRIC for the time periods of 0–5 and 5–10 years after diagnosis are also shown to allow comparisons with the relationships of the clinicopathologic parameters sooner after diagnosis. There is a clear excess of high nodal status, large tumour size and, to a lesser extent, high grade, that persisted in cases with a DR beyond 10 years compared to controls that is consistent between both the VLR and METABRIC datasets.

### Copy number alteration (CNA)

There was no significant difference in the percentage of genome with a CNA between cases and controls. Cases in METABRIC similarly showed no significant difference in CNAs from controls (Supplementary Figure 2). In METABRIC, tumours from patients showing recurrence by 5 years showed a significantly greater number of CNAs than those recurrence free beyond 5 years (*p* =  < 0.0001) but there was no difference for those recurring between 5 and 10 years and, consistent with the VLR data, those who were recurrence free in years 0–10 (*p* = 0.23) (Supplementary Figure 2). Thus, the importance of CNA for prognosis seems to be largely lost 10 years and possibly as early as 5 years after diagnosis.

Although the exomic analysis of the limited gene set conducted in VLR provided less sensitivity for gains and losses than the pan-genome analysis conducted in METABRIC, the overall patterns of gains and losses are similar and there are no significantly altered regions in the late recurrence METABRIC data. No chromosomal regions were altered significantly differently between cases (> 10 yr R) and controls (0–20 yr noR) in both VLR and METABRIC (Supplementary Figure 3). Similarly, there are no regions with significant differences after multiple correction between cases (0–5 yr R) and controls (0–10 yr noR) in METABRIC, in contrast to the many large chromosomal regions with highly significant differences for cases with earlier recurrences (Supplementary Figure 3).

### Mutation detection

**Fig. 2:**
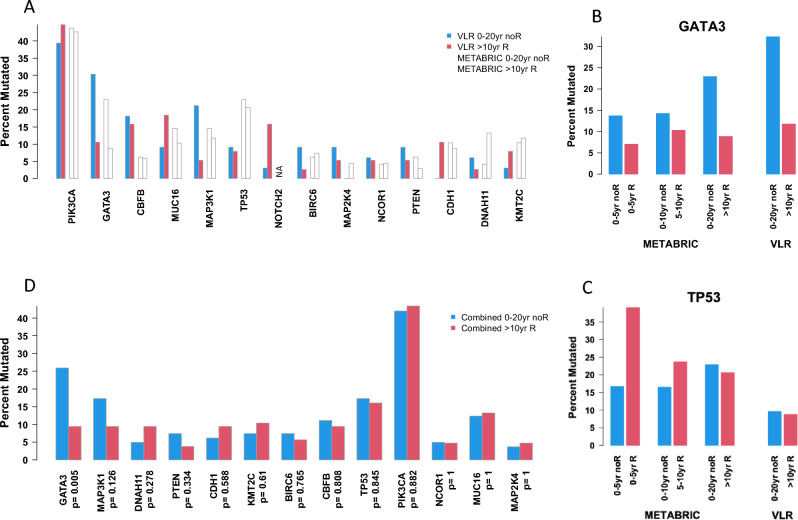
**A** Percentage of cases (red) and controls (blue) in VLR and METABRIC (> 10 yr R and > 20 yr noR) with a mutation in genes with at least 4 mutations overall in VLR; **B**, **C** in the combined VLR and METABRIC data for *GATA3* and *TP53*, respectively, in comparison with earlier time intervals for METABRIC; **D** in the combined VLR and METABRIC data for the 13 genes common to both analyses and with at least 4 mutations in the VLR cohort

Overall, there was no difference in somatic mutational burden between cases and controls. There were trends for greater numbers of *MAP3K1* and *GATA3* mutations in controls compared to cases (*p* = 0.07 and 0.07 respectively, Fig. 2A). This did not remain significant after correction for multiple testing but the pattern for both these genes was also seen in the METABRIC dataset, strikingly so for *GATA3*. The trend for greater proportion of *GATA3*-mutated tumours in the controls than in the cases with time to recurrence is evident in the METABRIC dataset (Fig. 2B) in contrast to *TP53* which showed highly significant differences in early recurrences (*p* < 0.00001) but not in later recurrences (> 10 yr *p* = 0.1 and > 20 yr *p* = 0.82) (Fig. 2C). *PIK3CA* was the most commonly detected mutation in both cases and controls and this was concordant with data from METABRIC. A combined analysis of the VLR and METABRIC data is shown in Fig. 2D and emphasizes the apparently protective effect of *GATA3* (*p* = 0.005) mutations for late DR with little difference in the incidence of the other mutations.

### Gene expression

**Fig. 3:**
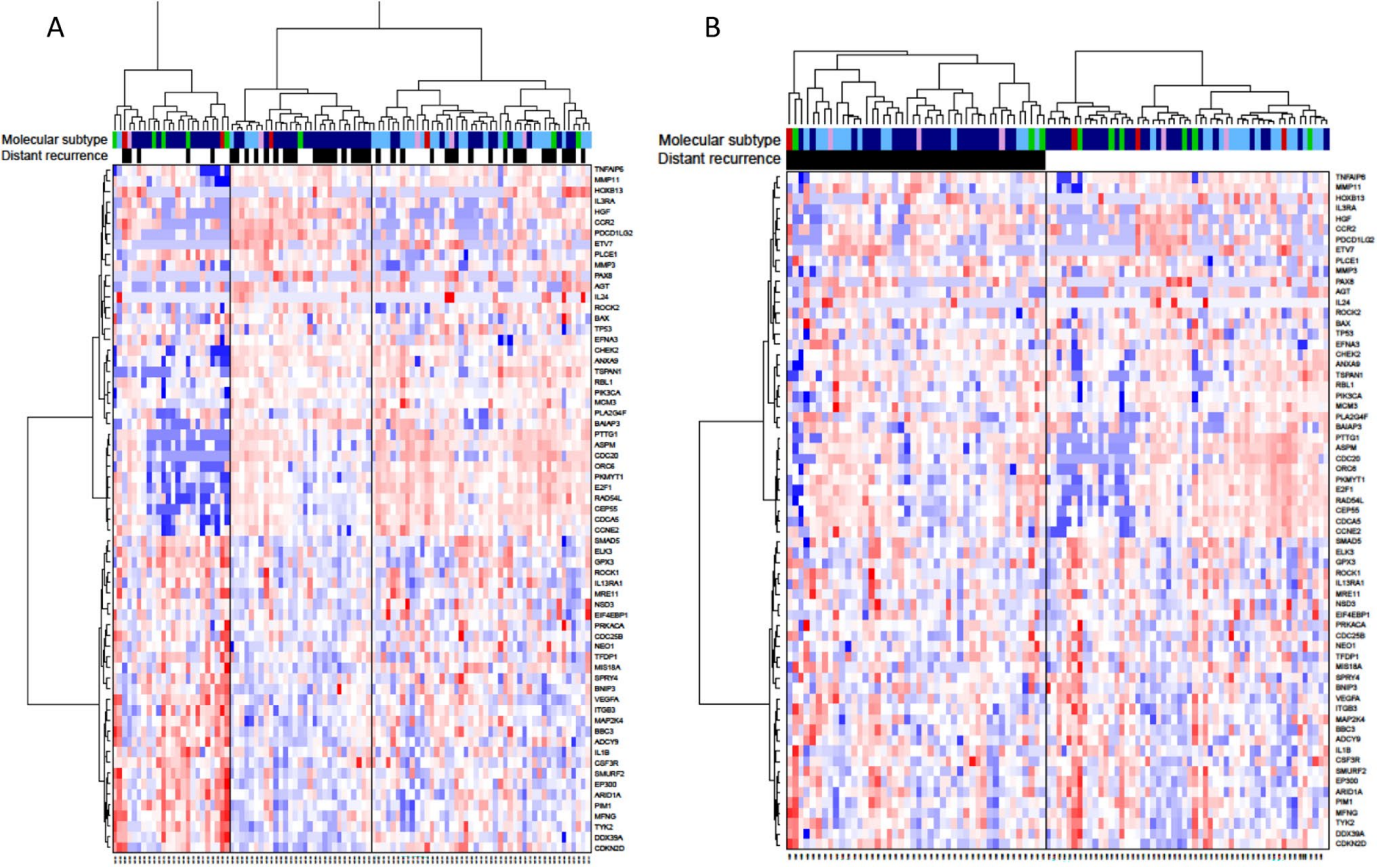
**A** Heatmap with unsupervised clustering of all samples analysed according to the patterns of expression of genes found to be significant and **B** with samples ordered by DR. Molecular subtype shown by coloured bars (dark blue—luminal A, pale blue—luminal B, pink—HER2-enriched, red—basal, green—normal-like). Recurrence is shown by black bar, non-recurrence shown by no bar

Figure 3 shows intrinsic molecular subtyping for VLR cases and controls. Distribution was largely as expected for an ER+ population with little difference between cases and controls. In particular, there was a similar proportion of cases and controls from within each of the luminal A and luminal B subtypes indicating no prognostic significance of these intrinsic subtypes beyond 10 years. The METABRIC data similarly showed no substantial differences in intrinsic subtypes between controls and cases after 10 years but did show the expected excess of luminal A tumours that were non-recurrent up to 5 years and between 5 and 10 years.

Of particular note, while both ESR1 and PGR showed higher expression in non-recurrent tumours in the first 5 years of follow-up in METABRIC, neither showed a significant difference after 10 years in either METABRIC or VLR (Supplementary Figure 4A and B). Conversely, proliferation (based on the average expression of the 18 proliferation genes of the PAM50 gene set [26]) showed significantly higher expression in patients with recurrences at 0–5 and 5–10 years in the METABRIC, but not after 10 years in both METABRIC and VLR (Supplementary Figure 4C).

Sixty-five individual genes were differentially expressed between cases and controls by univariate analysis (unpaired t-test; Supplementary Table 3, Supplementary Figure 5). After correction for multiple testing, none of these remained significant. Similarly, there were no significantly differentially expressed genes in METABRIC (> 10 years recurrence vs > 20 years no recurrence) after multiple correction.

Unsupervised hierarchical clustering of the samples according to the expression of all analysed genes showed the presence of 2 distinct clusters, which separated more according to their molecular subtype rather than their DR outcome status (Supplementary Figure 6).

Unsupervised hierarchical clustering of just the 65 significant genes highlighted three separate clusters of samples (Fig. 3A). The prevalence of recurrence was statistically significant between the 3 clusters (*χ*^2^ of 9.74, *p* = 0.0077). The most distinct of the 3 clusters contained a subset of 24 samples (extreme left-hand side of Fig. 3A). This cluster was enriched for luminal A and normal-like subtypes. Only 4 of the 24 patients (21%) and only 2 of the 14 (14%) luminal A in this cluster had a DR. These samples were characterized by high expression of immune-related genes and low expression of proliferation genes. In contrast to the first cluster, the second cluster was also dominated by luminal A subtype tumours but with higher DR rate: 20/45 (63%) of the patients in this cluster and 16/22 (73%) of those with a luminal A tumour had a DR. Cluster 2 had a gene expression pattern largely opposite to that of the first cluster. The third cluster was dominated by luminal B subtype and 20/45 patients had a DR. In general, this cluster showed less distinct gene expression groupings but of note there was a high expression of cell cycle and DNA replication-related genes. The second and third groups clustered more closely to one another than to the first, which itself had the most distinct pattern of gene expression. When clustering was ordered by DR status (Fig. 3B) there was no distinct gene expression pattern.

### Gene set enrichment analysis

Significantly reduced expression of gene sets involved with epigenetic regulation and cytokine and chemokine signalling were found to be associated with recurrence from the NanoString breast cancer 360 module analysis (Table 2). Genes involved in cell immune response and apoptosis were identified from the gene ontology analysis, apoptotic genes from the KEGG gene set analysis and cell cycle inhibition and DNA damage response genes from the Hallmarks gene set enrichment analysis. Following correction for multiple testing none of these remained significant.

**Table 2:** Gene set enrichment data. Gene ratio describes the ratio of genes within each gene set to total significant genes either up (a) or down (b) in cases

Gene set analysis	Gene set description	Gene Ratio	Bg Ratio	*p* value	*p*-adjust (FDR)	Gene name
*Down in cases*						
BC360	Epigenetic Regulation	3/31	18/743	0.04	0.34	ARID1A/BNIP3/NSD3
	Cytokine and Chemokine Signalling	5/31	48/743	0.04	0.34	CSF3R/IL13RA1/IL1B/TYK2/VEGFA
GO	Nuclear export	4/31	8/736	0.0002	0.26	DDX39A/IL1B/PRKACA/TP53
	Positive regulation of nuclear export	3/31	6/736	0.001	0.45	IL1B/PRKACA/TP53
	Mitochondrial outer membrane permeability	4/31	14/736	0.002	0.44	BBC3/BNIP3/TFDP1/TP53
KEGG	Huntington disease	4/27	16/587	0.004	0.31	BBC3/EP300/GPX3/TP53
	Human cytomegalovirus infection	8/27	69/587	0.009	0.31	ADCY9/EIF4EBP1/IL1B/ITGB3/PRKACA/ROCK1/TP53/VEGFA
HALLMARK	IL6/JAK/STAT signalling	6/22	31/451	0.002	0.07	CSF3R/IL13RA1/IL1B/ITGB3/PIM1/TYK2
	Reactive oxygen species	2/22	3/451	0.007	0.11	CDKN2D/GPX3
	Unfolded protein response	2/22	6/451	0.03	0.32	EIF4EBP1/VEGFA
*Up in cases*						
GO	DNA replication initiation	3/34	6/736	0.002	0.70	CCNE2/MCM3/ORC6
	Meiotic cell cycle	6/34	39/736	0.007	0.70	ASPM/CCNE2/CDC20/PKMYT1/PTTG/RAD54L
	DNA-dependent DNA replication	4/34	18/736	0.007	0.70	CCNE2/CHEK2/MCM3/ORC6
	Cell cycle	16/34	198/736	0.008	0.70	ASPM1/BAX/CCNE2/CDC20/CDCA5/CEP55/CHEK2/E2F1/HGF/MCM3/ORC6/PKMYT1/PTTG/RAD54L/RBL1/ROCK2
KEGG	Cell cycle	9/27	65/587	0.002	0.20	CCNE2/CDC20/CHEK2/E2F1/MCM3/ORC6/PKMYT1/PTTG/RBL1
	Inositol phosphate metabolism	2/27	7/587	0.04	0.76	PIK3CA/PLCE1
	Human T-cell leukaemia virus 1 infection	7/27	73/587	0.04	0.76	BAX/CCNE2/CDC20/CHEK2/E2F/PIK3CA/PTTG
HALLMARK	G2M checkpoint	7/20	52/451	0.004	0.09	CDC20/E2F1/MCM3/ORC6/PTTG1/RAD54L/RBL1

None of the breast cancer 360 modules were significantly increased in cases. From the gene ontology analysis, KEGG and Hallmark gene sets, gene sets associated with DNA replication and cell cycle progression were found to be significantly increased in expression in cases compared to controls (Table 2, Supplementary Figure 7). Following correction for multiple testing none of these remained significant.

## Discussion

A large metanalysis of clinical data from the EBCTCG reported that risk of DR and death from ER-positive breast cancer persists to at least 20 years despite at least 5 years’ endocrine therapy, even in low-stage disease where the risk of DR is close to linear until year 20 [2]. Understanding which patients have a continued risk of DR beyond 10 years when many would expect they were completely free of risk would enable targeting of late adjuvant therapy for those at high risk and reassurance for those at very low risk. The current study found that in both the VLR and METABRIC cohorts, clinical stage parameters most notably nodal status and tumour size measured at diagnosis continue to have prognostic significance beyond 10 years. This is consistent with the EBCTCG data and suggests that the CTS5, an integrative pathologic algorithm that estimates risk of DR between 5 and 10 years after diagnosis [3] might be extended to estimate risk beyond 10 years. If validated in this setting, use of CTS5 to estimate risk of recurrence beyond 10 years and potentially guide management decisions would have considerable value to clinicians and patients given the long potential survivorship of women with ER+ breast cancer.

An overarching message from the current work is that while there is clear, continued prognostic importance of these clinicopathologic factors beyond 10 years, this contrasts with the modest impact of molecular diagnostic features. This is despite some of those features, such as overall number of CNAs and intrinsic subtype, having a substantive association with recurrence risk in the METABRIC data earlier after diagnosis. For example, overall somatic mutational burden did not differ between cases and controls. However, *GATA3* showed fewer late DRs when mutated and the pattern was consistent between VLR and METABRIC. *GATA3* mutations are associated with lower pathological stage and improved survival outcomes [27, 28]. The current data extend these earlier observations to indicate that *GATA3* mutations have a persistent effect on prognosis even in patients who have not recurred after 10 years.

Regarding gene expression, on an individual gene level, following correction for multiple testing, no single gene was found to be significantly associated with cases or controls. However, observations from the hierarchical clustering based on the 65 genes expressed differentially between cases and controls were of substantial interest. While intrinsic subgrouping itself lost prognostic importance beyond 10 years, this clustering revealed 2 groups of largely luminal A tumours one of which was strongly associated with increased risk of late recurrence. Cell cycle and proliferation genes were upregulated in this subgroup, while immune-related genes and cell cycle inhibitors (eg CDKN2D) were down-regulated. Several of these genes also feature in gene set annotations whose expression was found to be significantly altered in cases compared to controls. In particular, hallmarks of proliferation were related to increased chance of late recurrence and immune-related hallmarks to decreased risk. This relationship with immune features is in contrast to our earlier observation that such immune-related genes, as well as the degree of lymphocytic infiltrate, were related to de novo resistance to the anti-proliferative effects of by aromatase inhibitors [29]. It would therefore appear that there may be a temporal relationship between ongoing risk of recurrence and immune features and risk of recurrence. Such reversal of molecular characteristics and prognosis with time is not unknown. ER-negativity itself is a marker of poor prognosis early in follow-up but a good prognosis feature later [30] and while high levels of ER are associated with good prognosis in the first 5 years after diagnosis, when most will be receiving endocrine therapy, they are associated with poorer prognosis beyond 5 years when endocrine treatment has stopped [6, 7]. Proliferation, however, continues to be important at least up to 10 years (Supplementary Figure 4C) [7] and VLR data indicate that this relationship continues to be associated with greater risk of recurrence beyond that point.

Few genes were analysed in both the RNA and DNA panels employed and therefore direct correlation of RNA expression and DNA copy number was limited. None of the 35 genes that overlapped showed statistically significant differential copy number or expression in cases or controls.

A strength of this study is its very long follow-up with available tissues, pathology conducted in a single institution and the use of the METABRIC dataset to confirm or refute findings. This study was, however, hampered by high attrition rates from the original number of cases and controls identified. This impaired the power of the study and contributed to the difficulty in identifying significant differences between cases and controls after using a conservative correction method but the availability of data from METABRIC allowed assessment of the consistency of differences showing a strong trend towards statistical significance in VLR.

This study did not match for clinical features which limits the power of the study to identify genes and pathways, independent of disease burden, that are associated with very late recurrence. However, large meta-analyses have identified that whilst clinical features are associated with late recurrence, even those with T1N0 cancers have an ongoing risk of recurrence to 20 years. Therefore, even without controlling for clinicopathological features, the associations of molecular biomarkers with very late recurrence identified in this study cannot be explained by their link to disease burden alone and are likely to represent a true effect that should be explored further.

Overall, the patient cohort in this study is representative of a true population of ER+ early breast cancer. The median age of the patients in our study was younger than that in a global population of breast cancer patients at diagnosis and is younger than that in METABRIC. This may reflect the Royal Marsden’s being a tertiary referral centre and might mean that some of our observations are unique to younger patients although that is not borne about by the similarity of our findings with those in METABRIC.

Our study was focussed on the need to distinguish between those with the potential for late recurrence and those who are cured from their cancer amongst women who have reached 10 years from diagnosis and did not compare clinical and molecular features of late recurring versus early recurring cancers. Currently, many women with high risk features will be advised to continue endocrine therapy to ten years owing to a significant (albeit modest) DFS benefit. However, risk of recurrence remains linear to at least 20 years and so some but not all women that are recurrence free at 10 years may benefit from continuing treatment. Further risk stratification at this point would be useful for the very large numbers of women reaching the 10-year disease-free timepoint.

The exploratory nature of the study leads to the observations being largely hypothesis generating, but as such, they lay a foundation for further investigation in the context of larger translational studies. A plausible hypothesis to explain our main observations is that the existence and extent of micrometastatic disease at surgery is determined by both tumour load, as exhibited by tumour size and nodal status, and molecular features that underpin behavioural aspects of the micrometastases. The tumour load factor is one that is “hard-wired” and lacks temporal significance since tumour excision removes any further biologic importance. In contrast the molecular features continue to impact on the behaviour and growth of micrometastases, with aggressive features leading to early relapse and the removal of those patients from the population at risk of late recurrence. The finding of the association of immune-related genes which may impede early recurrence with increased risk of late recurrence suggests that the use of checkpoint inhibitors might have a role in preventing late recurrence.

### Supplementary Information

Below is the link to the electronic supplementary material.

**Supplementary Figure 1.** Consort diagram of VLR study. **Supplementary Figure 2.** Boxplot showing overall CNA and gains (**A**), losses (**B**) between cases (red) and controls (blue) and overall CNAs (**C**) in cases and controls for both METABRIC and VLR. Tables shows the significance of the differences in expression between cases and controls based on Mann–Whitney tests. **Supplementary Figure 3.** Plots showing the percent of cases (red) and controls (blue) with copy number gains or losses for individual sites in the genome. The green dots represent the significance of the differences in CNAs between cases and controls based on fisher-exact tests. **Supplementary Figure 4.** Boxplots showing gene expression levels in cases (red) and controls (blue) for ESR1 (**A**), PGR (**B**) and the average of 18 PAM50 proliferation genes (**C**) for METABRIC and VLR cases and controls. Tables shows the significance of the differences in expression between cases and controls based on Mann–Whitney tests. **Supplementary Figure 5.** Volcano plot of gene expression. Dots represent genes (black—non significant, red—significant). Red dots to the right of the 0.0 point on the x axis represent genes expressed more highly in cases compared to controls. Red dots to the left of the 0.0 mark on the x axis represent genes expressed more highly in controls compared to cases. The further to the right or left of 0 indicates a greater magnitude of difference. The y axis shows increasing degree of significance. **Supplementary Figure 6.** Heatmap with unsupervised clustering of all samples analysed according to patterns of expression of genes found to be significant. Molecular subtype shown by coloured bars (dark blue—luminal A, pale blue—luminal B, pink—HER2 enriched, red—basal, green—normal like). Recurrence is shown by black bar, non-recurrence shown by no bar. **Supplementary Figure 7.** KEGG cell cycle gene set pathway showing genes expressed high in cases (red) and low in cases (green) (PDF 1378 KB)

**Supplementary Table 1.** Bed file showing genes in DNA targeted sequencing panel. **Supplementary Table 2.** Demographics of METABRIC groups 0–5 yr and 5–10 yr. **Supplementary Table 3.** 65 Genes differentially expressed in RNA analysis. **Supplementary Table 4.** List of total mutations by gene. **Supplementary Table 5.** List of mutations in each patient. **Supplementary Table 6.** Raw NanoString data for 98 VLR samples and 790 probes (XLSX 701 KB)

## Data Availability

Raw data from the DNA sequencing are located in Supplementary Tables 5 and 6 and RNA Nanostring data are located in Supplementary Table 7.
