# Supplementary material for: Clinical and molecular predictors of very late recurrence in oestrogen receptor-positive breast cancer patients
Source: Breast Cancer Res Treat. 2024 May 6;206(1):195–205. doi: 10.1007/s10549-024-07311-z (PMC11182842; doi:10.1007/s10549-024-07311-z)

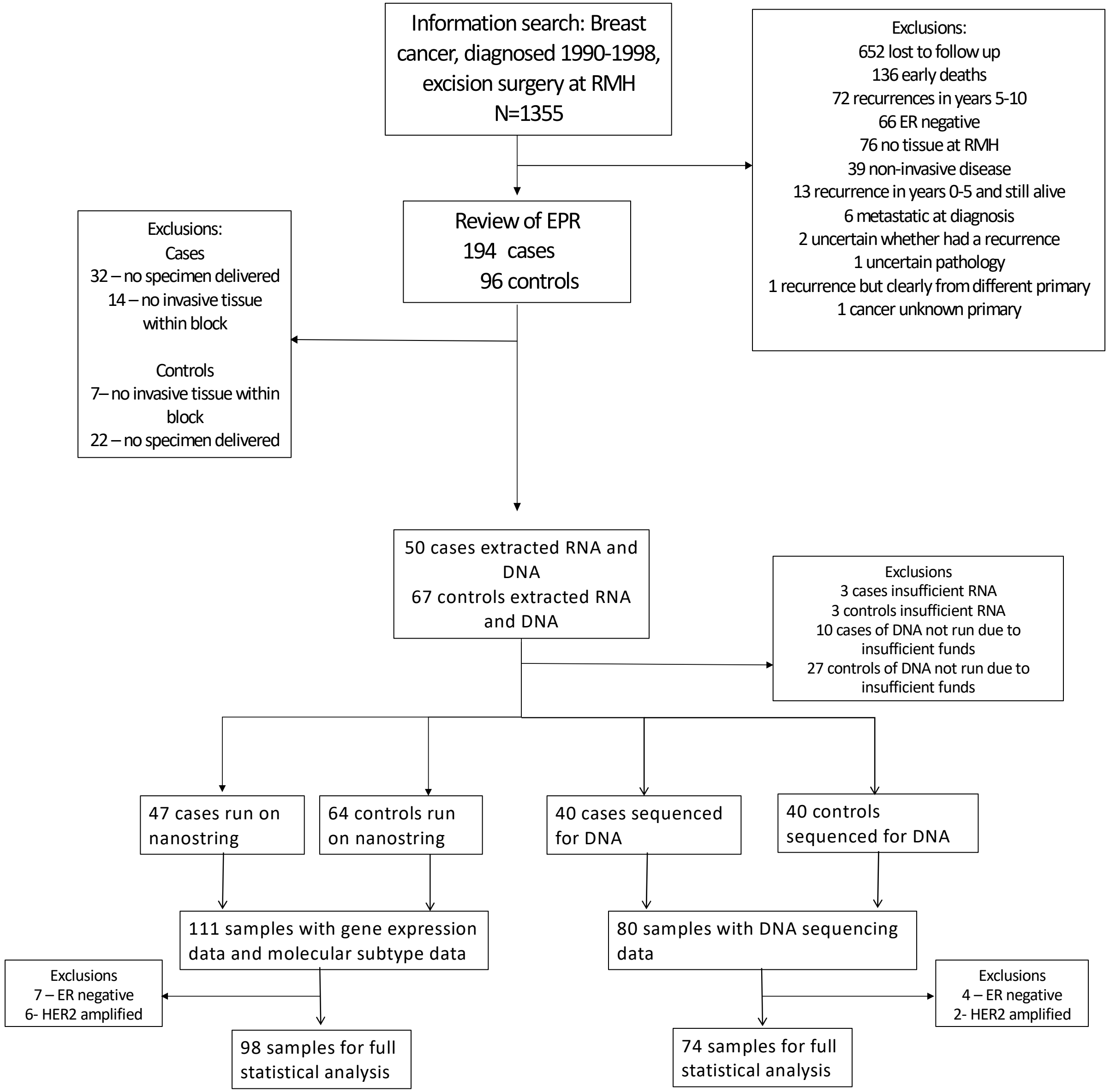

Supplementary Figure 1

Supplementary Figure 2

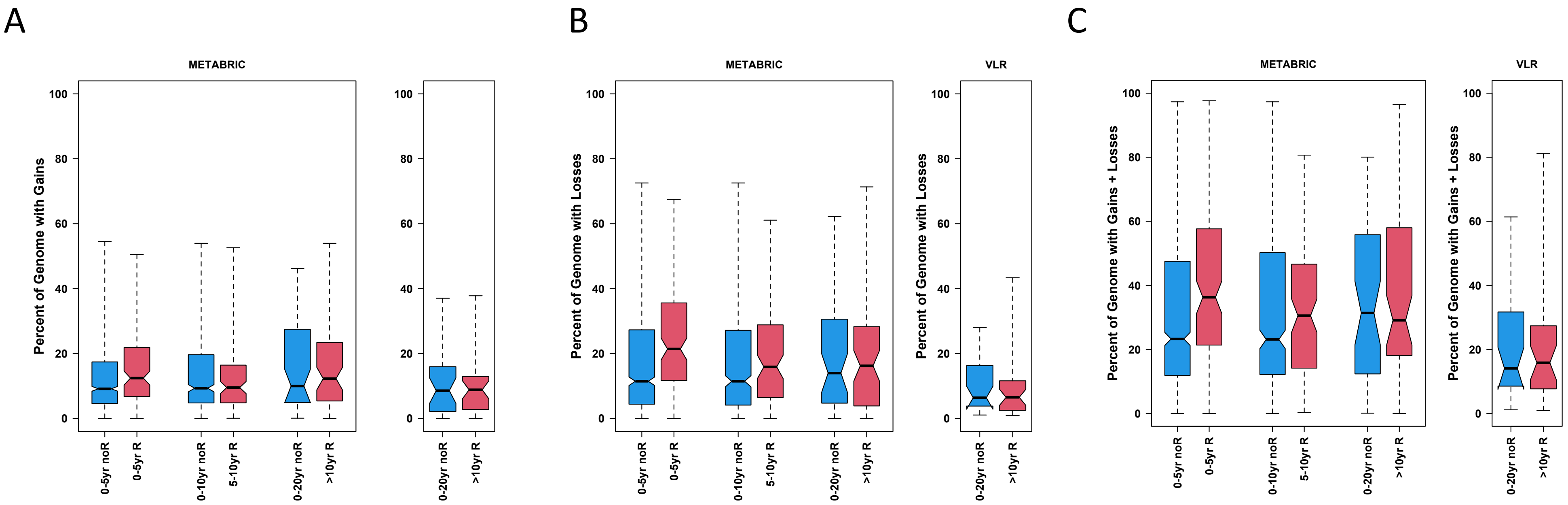

	P value (Mann-Whitney)
0-5yr noR vs 0-5yr R (METABRIC)	0.0005
0-10yr noR vs 5-10yr R (METABRIC)	0.84
0-20yr noR vs >10yr R (METABRIC)	0.60
0-20yr noR vs >10yr R (VLR)	0.65

	P value (Mann-Whitney)
0-5yr noR vs 0-5yr R (METABRIC)	< 0.00001
0-10yr noR vs 5-10yr R (METABRIC)	0.06
0-20yr noR vs >10yr R (METABRIC)	0.86
0-20yr noT vs >10yr R (VLR)	0.33

	P value (Mann-Whitney)
0-5yr noR vs 0-5yr R (METABRIC)	< 0.00001
0-10yr noR vs 5-10yr R (METABRIC)	0.33
0-20yr noR vs >10yr R (METABRIC)	0.65
0-20yr noR vs >10yr R (VLR)	0.55

Supplementary Figure 3

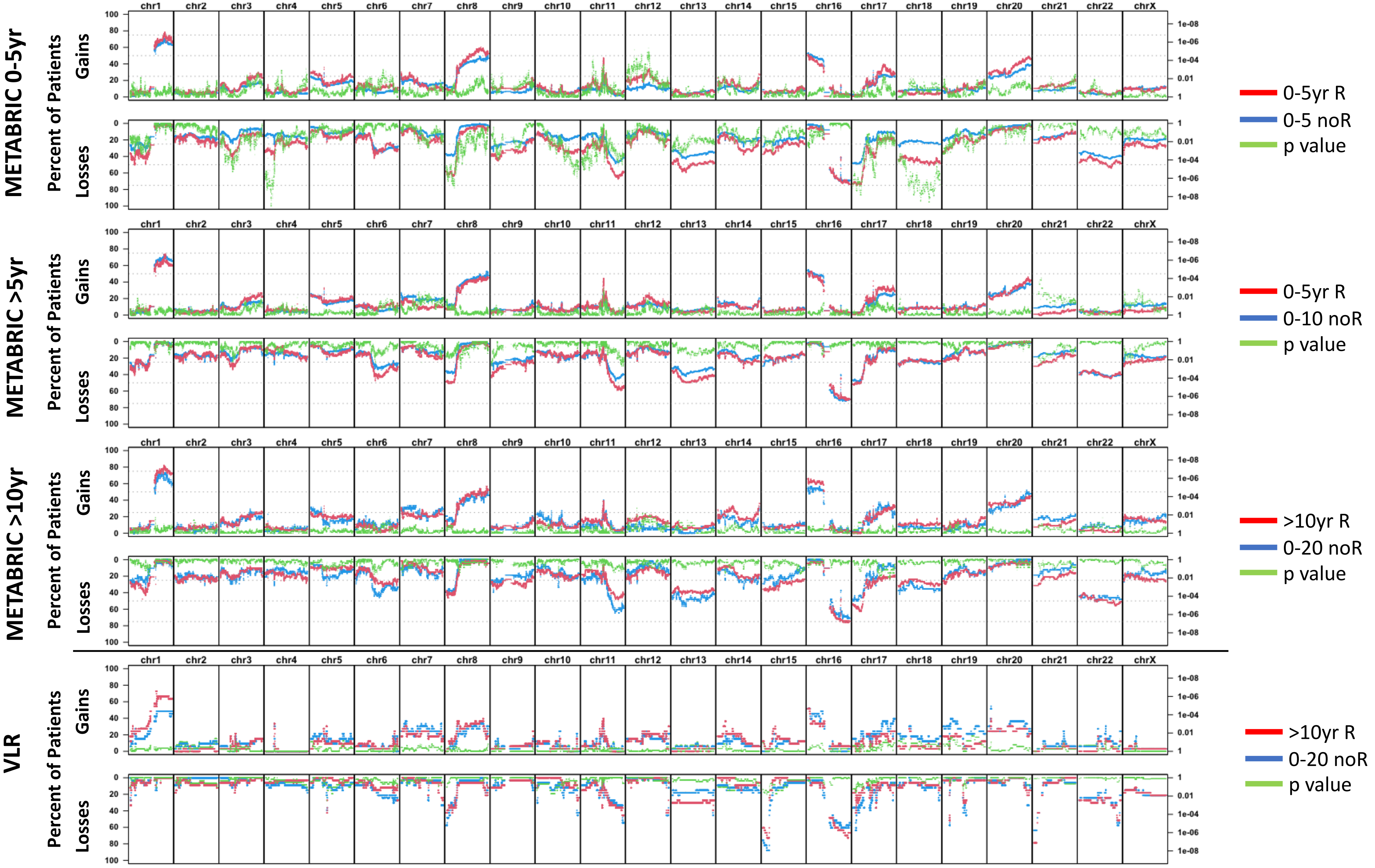

Supplementary Figure 4

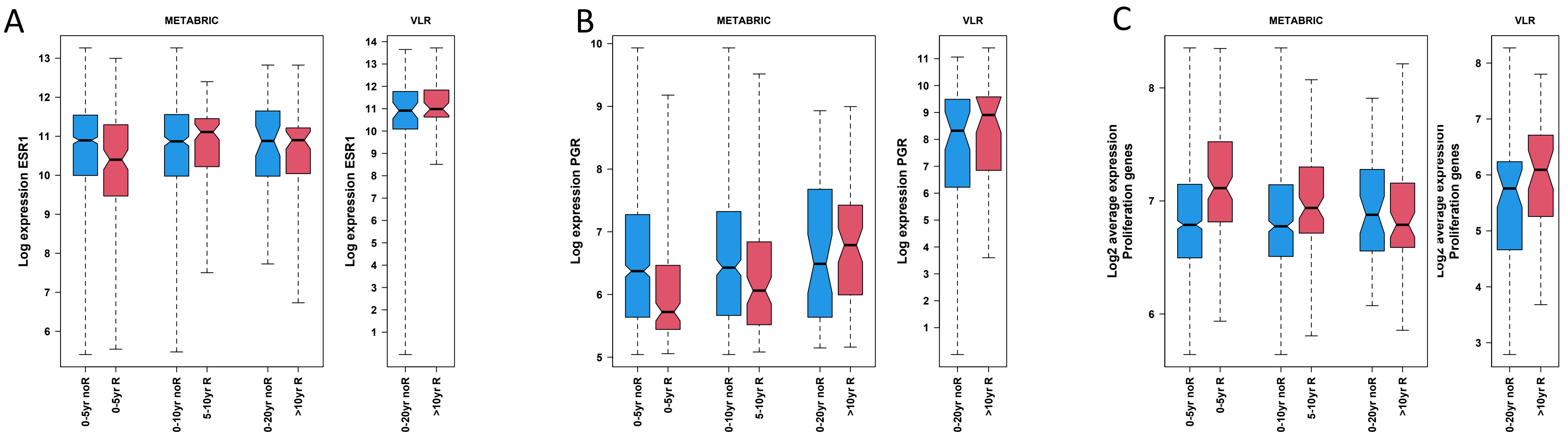

	P value (Mann-Whitney)
0-5yr noR vs 0-5yr R (METABRIC)	0.001
0-10yr noR vs 5-10yr R (METABRIC)	0.42
0-20yr noR vs >10yr R (METABRIC)	0.74
0-20yr noR vs >10yr R (VLR)	0.43

	P value (Mann-Whitney)
0-5yr noR vs 0-5yr R (METABRIC)	< 0.00001
0-10yr noR vs 5-10yr R (METABRIC)	0.006
0-20yr noR vs 0-10yr R (METABRIC)	0.28
0-20yr noR vs 0-10yr R (VLR)	0.21

	P value (Mann-Whitney)
0-5yr noR vs 0-5yr R (METABRIC)	< 0.00001
0-10yr noR vs 5-10yr R (METABRIC)	0.004
0-20yr noR vs 0-10yr R (METABRIC)	0.73
0-20yr noR vs 0-10yr R (VLR)	0.07

Supplementary Figure 5

A

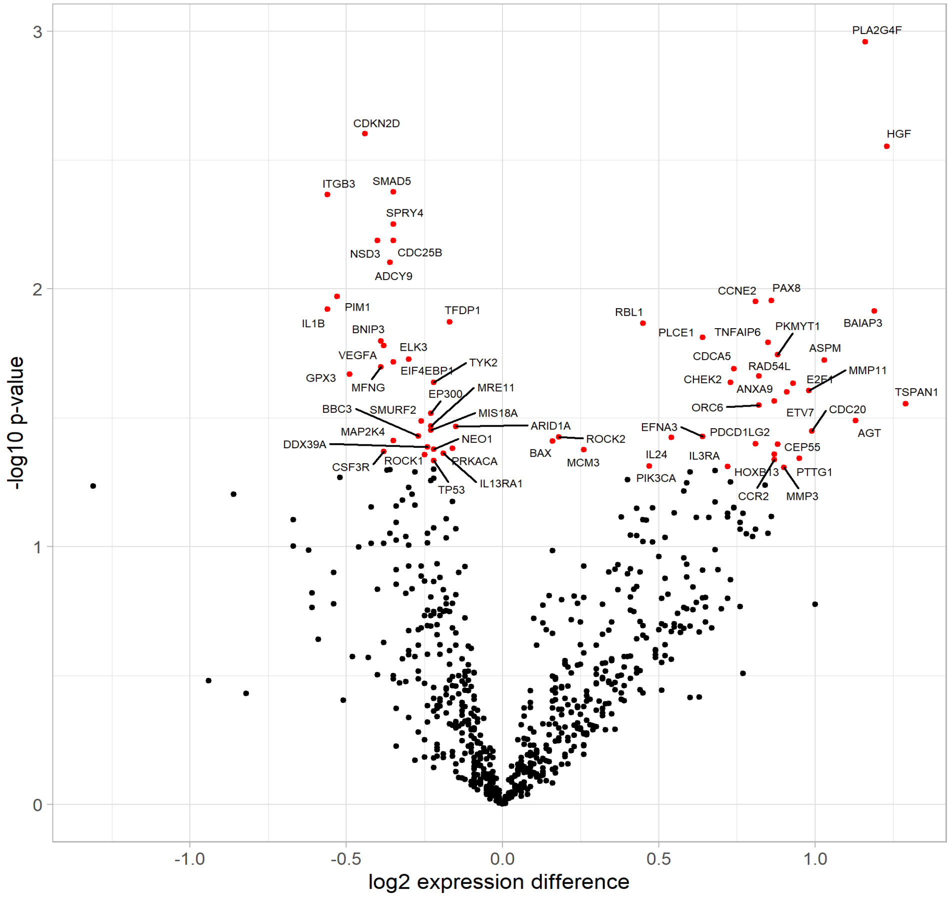

Supplementary Figure 6

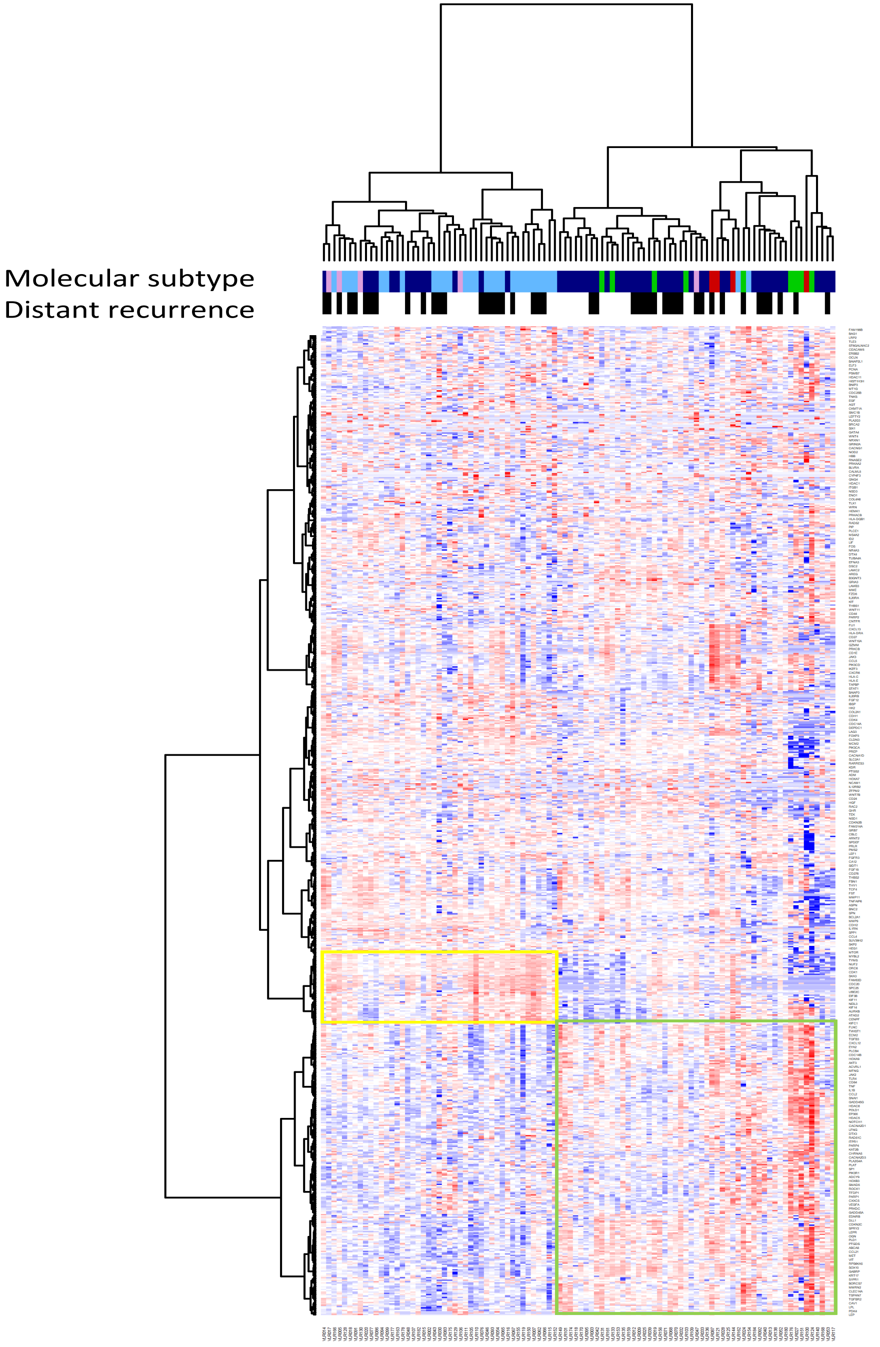

Supplementary Figure 7

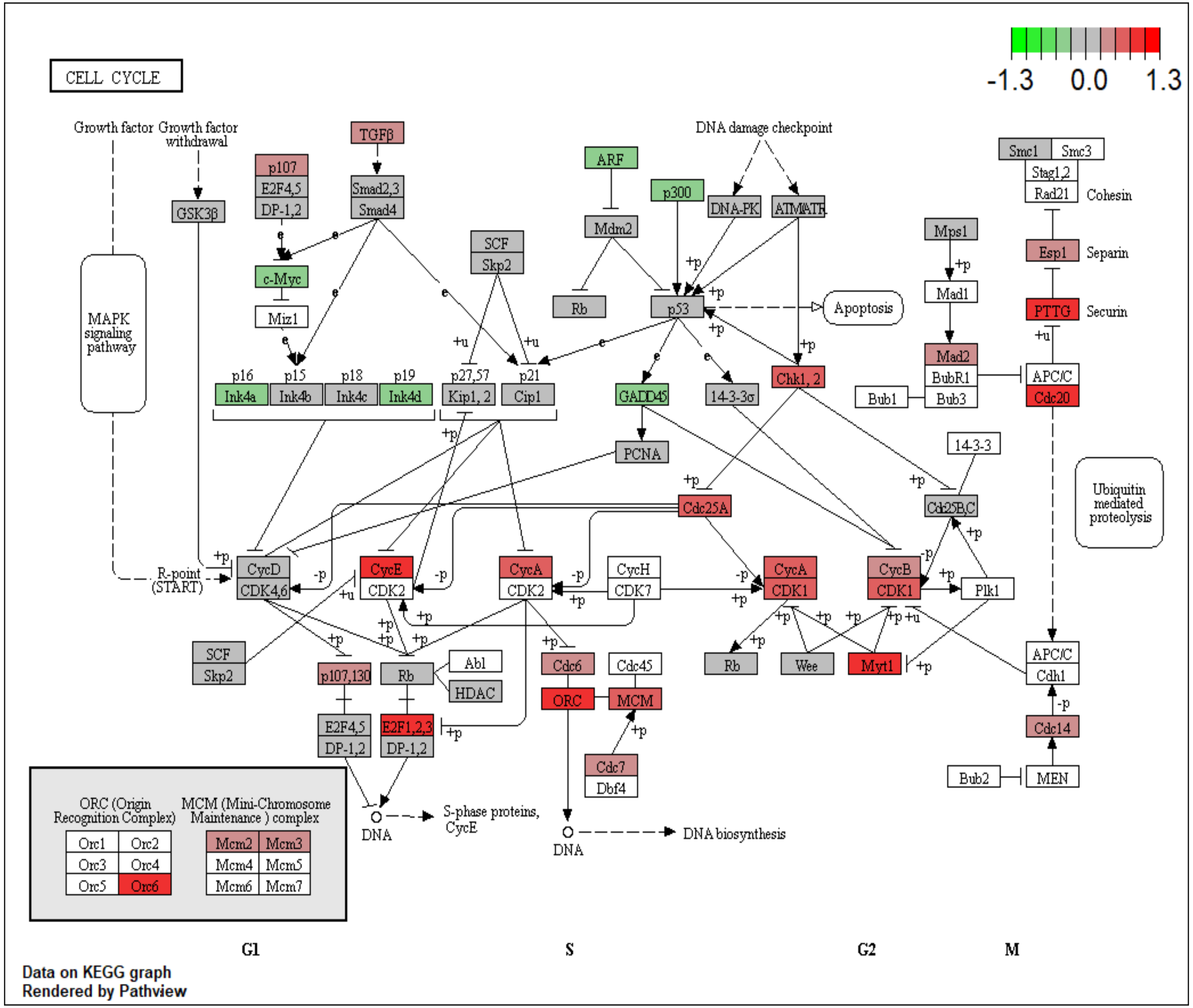

Supplement: Supplementary file 1 — Supplementary Figure 1. Consort diagram of VLR study. Supplementary Figure 2. Boxplot showing overall CNA and gains (A), losses (B) between cases (red) and controls (blue) and overall CNAs (C) in cases and controls for both METABRIC and VLR. Tables shows the significance of the differences in expression between cases and controls based on Mann–Whitney tests. Supplementary Figure 3. Plots showing the percent of cases (red) and controls (blue) with copy number gains or losses for individual sites in the genome. The green dots represent the significance of the differences in CNAs between cases and controls based on fisher-exact tests. Supplementary Figure 4. Boxplots showing gene expression levels in cases (red) and controls (blue) for ESR1 (A), PGR (B) and the average of 18 PAM50 proliferation genes (C) for METABRIC and VLR cases and controls. Tables shows the significance of the differences in expression between cases and controls based on Mann–Whitney tests. Supplementary Figure 5. Volcano plot of gene expression. Dots represent genes (black—non significant, red—significant). Red dots to the right of the 0.0 point on the x axis represent genes expressed more highly in cases compared to controls. Red dots to the left of the 0.0 mark on the x axis represent genes expressed more highly in controls compared to cases. The further to the right or left of 0 indicates a greater magnitude of difference. The y axis shows increasing degree of significance. Supplementary Figure 6. Heatmap with unsupervised clustering of all samples analysed according to patterns of expression of genes found to be significant. Molecular subtype shown by coloured bars (dark blue—luminal A, pale blue—luminal B, pink—HER2 enriched, red—basal, green—normal like). Recurrence is shown by black bar, non-recurrence shown by no bar. Supplementary Figure 7. KEGG cell cycle gene set pathway showing genes expressed high in cases (red) and low in cases (green) (PDF 1378 KB) [file 10549_2024_7311_MOESM1_ESM.pdf]
